# Effect of alpha-lipoic acid and caffeine-loaded chitosan nanoparticles on obesity and its complications in liver and kidney in rats

**DOI:** 10.1007/s00210-023-02507-4

**Published:** 2023-06-12

**Authors:** Hussein G. Sawie, Yasser A. Khadrawy, Mayada M. El-Gizawy, Hagar H. Mourad, Enayat A. Omara, Eman N. Hosny

**Affiliations:** 1 Medical Physiology Department, Medical Research and Clinical Studies Institute, National Research Centre, El-Behouth St, Giza, Egypt; 2 Pathology Department, Medical Research and Clinical Studies Institute, National Research Centre, Giza, Egypt

**Keywords:** Obesity, Liver, Kidney, α-Lipoic acid, Caffeine-chitosan nanoparticles, Oxidative stress

## Abstract

The present work investigated the effect of α-lipoic acid (ALA) and caffeine-loaded chitosan nanoparticles (CAF-CS NPs) on obesity and its hepatic and renal complications in rats. Rats were divided into control, rat model of obesity induced by high fat diet (HFD), and obese rats treated with ALA and/or CAF-CS NPs. At the end of the experiment, the activities of aspartate aminotransferase (AST), alanine aminotransferase (ALT), and alkaline phosphatase (ALP) and the levels of urea, creatinine, interleukin-1β (IL-1β), and tumor necrosis factor-α (TNF-α) were determined in the sera of animals. In addition, malondialdehyde (MDA), nitric oxide (NO), and reduced glutathione (GSH) were measured in hepatic and renal tissues. Renal Na^+^, K^+^-ATPase was assessed. The histopathological changes were examined in the hepatic and renal tissues. Obese rats showed a significant increase in AST, ALT, ALP, urea, and creatinine. This was associated with a significant increase in IL-1β, TNF-α, MDA, and NO. A significant decrease in hepatic and renal GSH and renal Na^+^, K^+^-ATPase activity was recorded in obese rats. Obese rats also showed histopathological alterations in hepatic and renal tissues. Treatment with ALA and/or CAF-CS NPs reduced the weight of obese rats and ameliorated almost all the hepatic and renal biochemical and histopathological changes induced in obese rats. In conclusion, the present findings indicate that ALA and/or CAF-CS NPs offered an effective therapy against obesity induced by HFD and its hepatic and renal complications. The therapeutic effect of ALA and CAF-CS NPs could be mediated through their antioxidant and anti-inflammatory properties.

## Introduction

Obesity is a common medical condition caused by accumulation of excess fat. It results from long-term imbalance between excessive caloric intake and low energy expenditure caused by poor dietary habits and insufficient physical activity (Hill et al. 2012). Obesity is defined as a body mass index (BMI) of 30 or higher, and it is responsible for approximately 2.8 million deaths each year (WHO 2021). Obesity is not only a metabolic disorder, but it is also a strong risk factor for a variety of life-threatening conditions, like inflammation, oxidative stress, insulin resistance, cancer, hypertension, heart diseases, stroke, fatty liver, and kidney diseases (Pi-Sunyer 2009; Kaur 2014; Petrie et al. 2018). Obesity has been linked to abnormal high fat deposition in hepatocytes (hepatic steatosis), which in turn results in fibrosis and cirrhosis through inducing lipid peroxidation and subsequent activation of stellate cells and collagen synthesis (Welch et al. 2022). Obesity has been also recognized as a major risk factor for chronic kidney disease and end-stage renal failure (Sun et al. 2020). Obesity has been correlated with higher levels of lipid accumulation in renal tissue. Several studies have indicated an association between renal lipid accumulation and activation of pro-inflammatory cytokines like interleukin-6 and tumor necrosis factor-α (TNF-α) that play a key role in mediating cellular injury and renal dysfunction (D'Agati et al. 2016; Chen et al. 2019).

High fat diet (HFD) is the most useful model in studying obesity and metabolic syndrome in rodents (Hariri and Thibault 2010). The main advantages of using HFD-induced model of obesity lie in its physiological properties and in its similarity to human disease etiology (Buettner et al. 2007). HFD-induced obesity is characterized by overconsumption of HFDs due to their low satiating effects and the high efficiency of dietary fat in being stored in the body as well as the alterations in the hormones involved in energy balance, such as HFD-induced hyperinsulinaemia and hyperleptinaemia and accompanied by insulin and leptin resistance, and lowered suppression of ghrelin secretion following HFDs (Hariri and Thibault 2010).α-Lipoic acid (thioctic acid), a fat- and water-soluble powerful antioxidant, is a naturally occurring dithiol compound derived from octanoic acid (Ghibu et al. 2008). α-Lipoic acid (ALA) can be found in tomato, Brussels sprouts, broccoli, spinach, rice bran, red meat, and entrails including heart, kidney, and liver (Shay et al. 2009). An increasing number of studies have revealed that ALA exhibits a significant therapeutic and protective effect against many diseases associated with oxidative stress and inflammation, including neurodegenerative diseases, diabetes, cardiovascular disease, cancer, chronic liver diseases, hypothyroidism, and kidney diseases (Abdel-Zaher et al. 2008; Wongmekiat et al. 2013; Salehi et al. 2019; Khadrawy et al. 2022). The antioxidant property of ALA may be attributed to its ability to scavenge free radicals (Bast and Haenen 2003), its ability to enhance the activities of antioxidant enzymes (Gomes and Negrato 2014), and its ability to regenerate endogenous antioxidants including reduced glutathione (GSH), vitamin E, and vitamin C (Rochette et al. 2015). ALA acts also as an essential cofactor for mitochondrial pyruvate dehydrogenase and α-ketoglutarate dehydrogenase, both of which are important enzymes in cellular energy metabolism (Shay et al. 2009). It has been reported that ALA could be an effective treatment of obesity by increasing energy expenditure, reducing food intake, preventing lipid accumulation in adipose and non-adipose tissues, increasing mitochondrial biogenesis, and stimulating lipolysis and fat oxidation (Fernández–Galilea et al. 2013). Moreover, Ziegler (2004) have found that ALA has potential beneficial effects against obesity-induced complications including metabolic syndrome, insulin resistance, type II diabetes, and vascular damage. Although the wide safety margin of ALA, some rare adverse effects have been reported with very high doses of ALA (Emir et al 2018) and in case of thiamine deficiency (Najm 2012).

Caffeine (1,3,7-trimethylxanthine) is a naturally occurring purine alkaloid found in coffee, cola, cocoa, and tea (Heckman et al. 2010). Several studies reported that caffeine significantly reduced body fat mass by increasing lipolysis and inhibiting fat accumulation (Kobayahi-Hattori et al. 2005; Sugiura et al. 2012). Moreover, an increasing number of studies have demonstrated the beneficial effects of caffeine against experimentally induced hepatic and renal toxicities (Khazaei et al. 2012; Cachón et al. 2017; Anwar and Laila 2022). The ameliorative activity of caffeine could be attributed to its ability to scavenge reactive oxygen species (Devasagayam et al. 1996), its anti-inflammatory properties (Kang et al. 2012), and its ability to attenuate fibrotic processes (Arauz et al. 2014).

Caffeine is highly soluble but has rapid absorption, rapid distribution, and complete bioavailability, as well as its ability to cross lipid membranes (Arnaud 2011). After 15–120 min, the oral plasma concentration reaches its peak. Caffeine plasma half-life ranges between 3 and 5 h as a result of its rapid distribution and elimination (White et al. 2016). This necessitates its repeated administration throughout the day in order to maintain adequate blood concentration (Teixeira 2009). As a result, it is preferable to have a controlled sustained release formula that reduces administration frequency while maintaining adequate therapeutic drug levels and increasing patient compliance. Chitosan nanoparticles have been successfully discovered as drug carriers, with the potential to enhance bioavailability, efficacy, and the capacity to achieve sustained drug release (Garg et al. 2019). The use of caffeine-loaded chitosan nanoparticles (CAF-CS NPs) has the advantage of the long-lasting release of caffeine which may enhance its therapeutic effects and reduce its adverse effects. CAF-CS NPs were used topically (Abosabaa et al. 2021); however, no studies were carried out investigating its parenteral administration.

Accordingly, the present study was conducted to evaluate the therapeutic effects of ALA and/or CAF-CS NPs against obesity and its hepatic and renal complications generated in rats fed on a high fat diet. ALA was co-administered with CAF-CS NPs to investigate the synergistic effect between them.

## Materials and methods

### Animals

Fifty male Wistar albino rats obtained from Animal House Colony of National Research Centre, Giza, Egypt, were used in the present study. Their weights ranged from 120 to 150 g. Animals were housed in stainless steel cages with ad libitum access to standard laboratory diet and tap water. They were placed in a temperature-controlled (20–25 °C) and artificially illuminated (12-h dark/light cycle) room free of any chemical contamination. Animal procedures were approved by the Ethics Committee of the National Research Centre (with ethical approval number of 20,149) and were performed in compliance with the recommendations of the National Institutes of Health Guide for Care and Use of Laboratory Animals (publication no. 85–23, revised 1985).

### Chemical and drugs

α-Lipoic acid (ALA) was purchased from EVA Pharma for Pharmaceuticals and Medical Appliances, Cairo, Egypt. Caffeine and chitosan were obtained from Sigma-Adrich, Germany. The method used in the preparation of caffeine-loaded chitosan nanoparticles (CAF-CS NPs) depended on the method described by Sahudin et al. (2018). In this method, nanoparticles (NPs) were prepared by inducing the gelation of chitosan (CS) solutions with the cross-link agent sodium tripolyphosphate (TPP). 0.2% low molecular weight chitosan solution was prepared in 1% acetic acid while 0.1% TPP was dissolved in distilled water. CS NPs were spontaneously formed upon addition of TPP into CS solution. Caffeine was dissolved in 0.1% TPP solution. CAF-CS NPs were formed when TPP-containing drug solution is added dropwise into 25 ml of CS solution with magnetic stirring for 30 min at room temperature. The resulting CAF-CS NPs were subjected to ultra-sonication for a few minutes. The CAF-CS NPs were then separated from their suspension by centrifugation.

### Transmission electron microscopy (TEM) imaging

The shape and average size of the CAF-CS NPs were detected using transmission electron microscopy (TEM). A small quantity of CAF-CS NPs solution (1 mg/ml) was placed on surface of a TEM grid. After a few minutes of incubation, excess fluid was removed with filter paper and the grid surface was air-dried at room temperature. It was then loaded into the transmission electron microscope (JEM-HR-2100 electron microscope, Japan) at total magnification 6.00 kx and accelerating voltage 200 kV.

### Induction of rat model of obesity

Rat model of obesity was induced by feeding the rats on a high fat diet (HFD) (45% fat, 41% carbohydrate, and 19% protein) for 20 weeks (McNeilly et al. 2011). The body mass index (BMI) was used as an indicator for obtaining obese rats. Body weight and body length measurements were used to calculate BMI. Body weight and body length of rats were measured using a weighing scale and tape rule, respectively. The BMI was determined using the formula (Novelli et al. 2007):

$$\mathrm{BMI}=\frac{\text{Body weight (g)}}{{\text{Body length}}^{2}\text{ (}{\text{cm}}^{2}\text{)}}$$

Obese rats were defined by a BMI of greater than 0.68 g/cm^2^ as previously described by Novelli et al. (2007). Rats that did not meet the obesity BMI in the experimental group after 20 weeks of HFD were excluded from the study. However, all the rats in the experimental group attained the target BMI and were all included.

### Experimental design

At the beginning of the experiment, the rats were divided into five groups of ten rats each. Rats in the control group were fed a standard diet (12% fat, 67% carbohydrate, and 21% protein) until the end of the experiment. The rest of the animals were used to induce rat model of obesity by feeding the rats on a HFD for 20 weeks according to McNeilly et al. (2011). Then, these animals were subdivided into rat model of obesity treated daily with saline solution for 4 weeks, rat model of obesity treated daily with alpha ALA (100 mg/kg, by gavage) (Perera et al. 2011) for 4 weeks, rat model of obesity injected daily with CAF-CS NPs (20 mg/kg, intraperitoneally) (Horvath et al. 2022) for 4 weeks, and rat model of obesity treated daily with ALA and CAF-CS NPs 1 h between each treatment for 4 weeks.

### Preparation of samples

At the end of the experiment, animals of all groups were sacrificed by sudden decapitation. Then, blood samples were collected and centrifuged at 3000 rpm for 15 min at 4 °C to separate sera which were stored at − 20 °C until the measurement of interleukin-1β (IL-1β), tumor necrosis factor-α (TNF-α), and parameters of hepatic and renal function. The liver and kidney of each rat were quickly excised and washed with saline to get rid of any blood. A part of the liver tissue and the left kidney were fixed immediately in 10% formalin solution for histological examination. Each of the liver tissue and the right kidney was weighed and homogenized in Tris–HCl buffer (pH 7.4). The homogenate was centrifuged at 3000 rpm for 10 min at 4 °C, and the supernatant was stored at − 20 °C until the determination of Na^+^, K^+^-ATPase and oxidative stress parameters.

### Assessment of liver functions

The activities of aspartate aminotransferase (AST), alanine aminotransferase (ALT), and alkaline phosphatase (ALP) were determined in the serum according to the methods described by Reitman and Frankel (1957), and Belfield and Goldberg (1971), respectively, using kits supplied by Spectrum-diagnostic Company (Cairo, Egypt).

### Assessment of kidney functions

The serum levels of urea and creatinine were estimated spectrophotometrically according to the methods described by Fawcett and Scott (1960), and Schirmeister et al. (1964), respectively, using kits purchased from Spectrum-diagnostic Company (Cairo, Egypt).

### Determination of lipid peroxidation

Lipid peroxidation in terms of malondialdehyde (MDA) formation was estimated in hepatic and renal tissue homogenates according to the method of Ruiz-Larrea et al. (1994). In this method, MDA reacts with thiobarbituric acid producing thiobarbituric acid reactive substance, a pink colored complex, which can be measured spectrophotometrically at 532 nm.

### Determination of nitric oxide

Nitric oxide (NO) was measured spectrophotometrically in the hepatic and renal tissues according to the method described by Montgomery and Dymock (1961). This method depends on the measurement of endogenous nitrite concentration as an indicator of NO production. It depends on the addition of Griess reagent which converts nitrite into a deep purple azo compound whose absorbance is read at 540 nm.

### Determination of reduced glutathione

Reduced glutathione (GSH) was determined in hepatic and renal tissue homogenates according to the method of Beutler et al. (1963) using Ellman’s reagent. The procedure is based on the reduction of Ellman’s reagent by SH groups to produce 5,5′-dithiobis (2-nitrobenzoic acid) which has an intense yellow color that is measured spectrophotometrically at 412 nm.

### *Determination of Na*^+^*, K*^+^*-ATPase activity*

Na^+^, K^+^-ATPase activity was measured in renal tissue homogenates according to the method described by Tsakiris et al. (2000). Na^+^, K^+^-ATPase activity was calculated as the difference between total ATPase activity (Na^+^, K^+^-ATPase and Mg-ATPase activity) and Mg-ATPase activity. The results were expressed as μmol Pi/min/g kidney tissue.

### Determination of serum interleukin-1β

Interleukin-1β (IL-1β) was measured in the serum using rat IL-1β ELISA kit obtained from Sino Gene Clon Biotech Co., Ltd, Hang Zhou, China, according to the company method instruction. The developed color was read at 450 nm using a microtiter plate reader. The concentration was then calculated from the standard curve. The concentration of IL-1β was expressed in ng/l.

### Determination of serum tumor necrosis factor-α (TNF-α)

Tumor necrosis factor-α (TNF-α) was measured in the serum using rat TNF-α ELISA kit obtained from Sino Gene Clon Biotech Co., Ltd, Hang Zhou, China, according to the company method instruction. The developed color was read at 450 nm using a microtiter plate reader. The concentration was then calculated from the standard curve. The concentration of TNF-α was expressed in ng/l.

### Histopathological examination

The liver and kidney of different groups were dissected out and grossly inspected for any changes and fixed immediately in 10% formalin solution. Sections (4 µm thick) were cut from paraffin blocks. The sections were stained by hematoxylin and eosin (H&E) and then examined with a light microscope for histological changes.

### Statistical analysis

The data were expressed as means ± S.E.M. Statistical Package for Social Sciences (SPSS) software (version 16) was used for all statistical calculations. Statistical difference between the groups under investigation was carried out using one-way analysis of variance (ANOVA) followed by Duncan as post hoc test. The difference was considered significant at *P*-value ≤ 0.05.

## Results

### TEM image

**Fig. 1:**
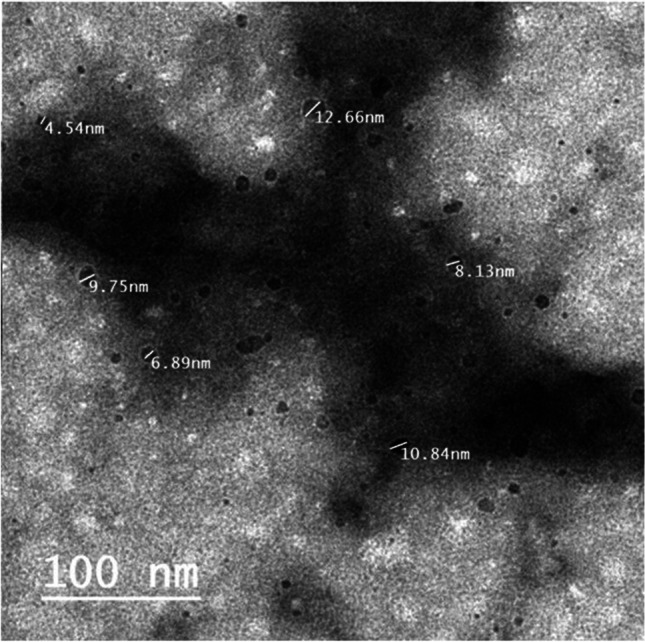
TEM image of the prepared CAF-CS NPs

As shown in Fig. 1, TEM imaging of caffeine-loaded chitosan nanoparticles (CAF-CS NPs) showed that the average particle size was 4–12 nm.

### Body mass index

**Fig. 2:**
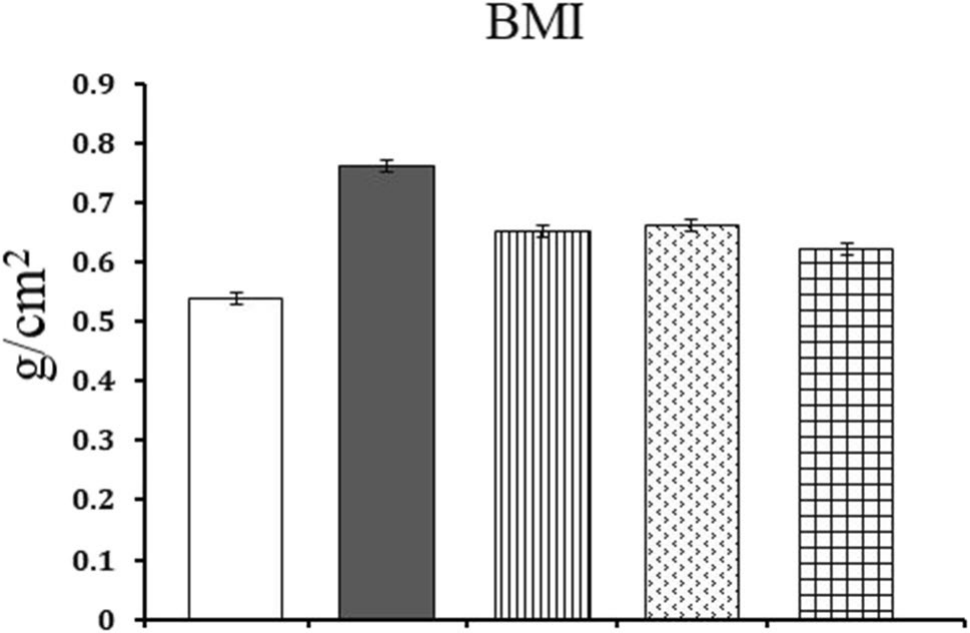
Effect of daily treatment with alpha-lipoic acid (ALA) (100 mg/kg) and/or caffeine-loaded chitosan nanoparticles (CAF-CS NPs) (20 mg/kg) for 30 days on body mass index (BMI).  Control**.**  Rat model of obesity.  Rat model of obesity treated with ALA.  Rat model of obesity treated with CAF-CS NPs.  Rat model of obesity treated with ALA and CAF-CS NPs

The body mass index (BMI) of control rats was 0.54 gm/cm^2^. However, in rat model of obesity, BMI recorded 0.76 g/cm^2^. When obese rats were treated with ALA and/or CAF-CS NPs, BMI reduced to 0.65 g/cm^2^, 0.66 g/cm^2^, and 0.62 gm/cm^2^ respectively (Fig. 2).

### Liver function biomarkers

Data presented in Table 1 show that HFD induced a significant increase in the serum activities of AST by + 92.13% (from 18.55 ± 1.90 to 35.64 ± 6.32, *P* = 0.000), ALT by + 193.27% (from 15.46 ± 2.59 to 45.34 ± 2.97, *P* = 0.000), and ALP by + 164.16% (from 76.47 ± 13.73 to 202.0 ± 27.05, *P* = 0.001) compared to the normal control values. Treatment with ALA and/or CAF-CS NPs returned the increased ALT and ALP activities caused by HFD to control rats. Concerning AST activity, only the treatment with ALA restored the serum AST to nearly control like values, while treatment with CAF-CS NPs alone or in combination with ALA failed to produce a similar effect recording + 116.23% and + 104.26% respectively more than the control value.

**Table 1:** Effect of daily treatment with alpha-lipoic acid (ALA) (100 mg/kg for 30 days) and/or caffeine-loaded chitosan nanoparticles (CAF-CS NPs) (20 mg/kg for 30 days) on the serum activity of aspartate aminotransferase (AST), alanine aminotransferase (ALT), and alkaline phosphatase (ALP) in rat model of obesity induced by feeding rats on a high fat diet (HFD)

	Control	Rat model of obesity	%D	ALA treated rats	%D	CAF-CS NPs treated rats	%D	ALA + CAF-CS NPs treated rats	%D	*P*-value
AST (U/l)	18.55^a^ ± 1.90	35.64^b^ ± 6.32	+ 92.13	20.79^a^ ± 1.59	+ 12.08	40.11^b^ ± 4.81	+ 116.23	37.89^b^ ± 2.29	+ 104.26	0.000
ALT (U/l)	15.46^a^ ± 2.59	45.34^b^ ± 2.97	+ 193.27	11.71^a^ ± 0.93	− 24.26	17.70^a^ ± 1.77	+ 14.49	12.52^a^ ± 0.76	− 19.02	0.000
ALP (U/l)	76.47^a^ ± 13.73	202.0^b^ ± 27.05	+ 164.16	100.04^a^ ± 12.74	+ 30.82	91.69^a^ ± 23.57	+ 19.90	122.97^a^ ± 19.06	+ 60.81	0.001

Values represent mean ± S.E

*%D*, % difference with respect to control values

Different letters indicate significantly different means at *P*-value < 0.05

Same letters indicate nonsignificant changes

### Kidney function biomarkers

The present results revealed that HFD resulted in a significant increase in serum levels of urea by + 39.43% (from 18.59 ± 1.92 to 25.92 ± 1.41, *P* = 0.001) and creatinine by + 120.51% (from 0.39 ± 0.04 to 0.86 ± 0.06, *P* = 0.000) compared to the control values. However, treatment with ALA and/or CAF-CS NPs succeeded in returning the significant increase in urea and creatinine levels induced by HFD to normal control values (Table 2).

**Table 2:** Effect of daily treatment with alpha-lipoic acid (ALA) (100 mg/kg for 30 days) and/or caffeine-loaded chitosan nanoparticles (CAF-CS NPs) (20 mg/kg for 30 days) on the serum level of urea and creatinine in rat model of obesity induced by feeding rats on a high fat diet (HFD)

	Control	Rat model of obesity	%D	ALA-treated rats	%D	CAF-CS NPs-treated rats	%D	ALA + CAF-CS NPs-treated rats	%D	*P*-value
Urea (mg/dl)	18.59^a^ ± 1.92	25.92^b^ ± 1.41	+ 39.43	20.93^a^ ± 1.04	+ 12.59	22.14^a^ ± 0.70	+ 19.10	18.50^a^ ± 1.25	− 0.48	0.001
Creatinine (mg/dl)	0.39^ac^ ± 0.04	0.86^b^ ± 0.06	+ 120.51	0.34^a^ ± 0.05	− 12.82	0.35^a^ ± 0.04	− 10.26	0.51^c^ ± 0.06	+ 30.77	0.000

Values represent mean ± S.E

*%D*, % difference with respect to control values

Different letters indicate significantly different means at *P*-value < 0.05

Same letters indicate non-significant changes

### Oxidative stress parameters

**Fig. 3:**
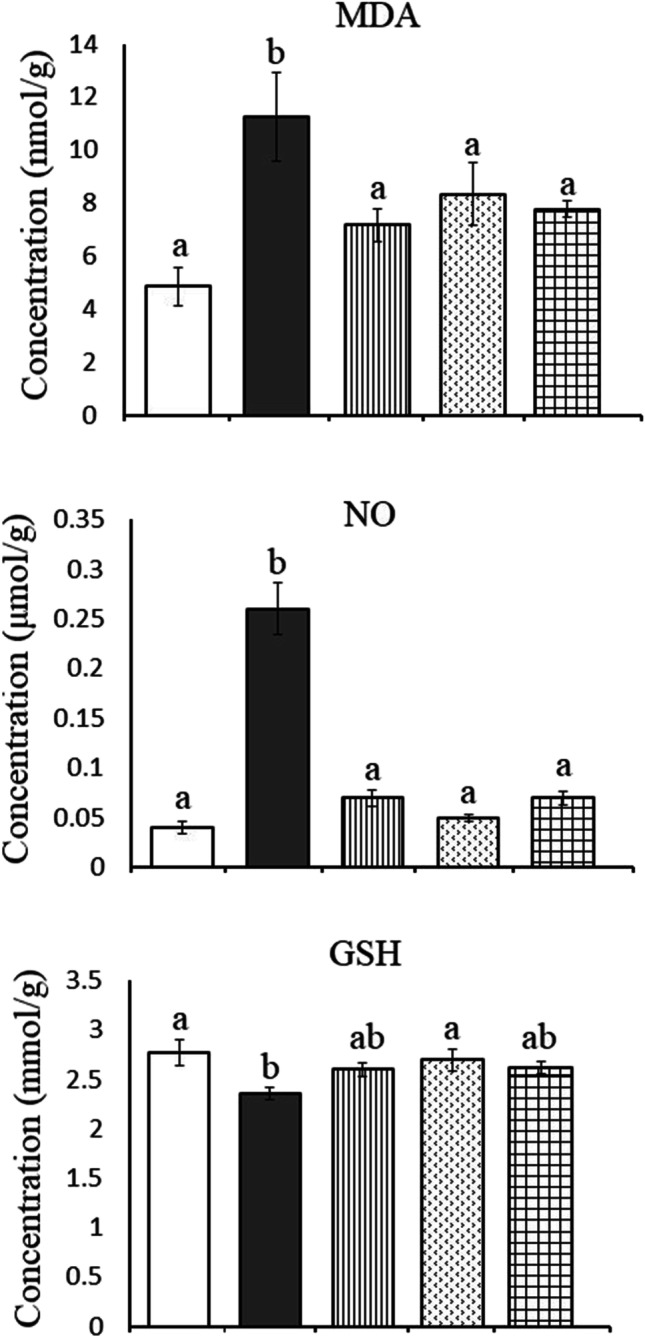
Effect of daily treatment with alpha-lipoic acid (ALA) (100 mg/kg) and/or caffeine-loaded chitosan nanoparticles (CAF-CS NPs) (20 mg/kg) for 30 days on the levels of malondialdehyde (MDA), nitric oxide (NO), and reduced glutathione (GSH) in the liver of rat model of obesity induced by feeding rats on a high fat diet (HFD).  Control**.**  Rat model of obesity.  Rat model of obesity treated with ALA.  Rat model of obesity treated with CAF-CS NPs.  Rat model of obesity treated with ALA and CAF-CS NPs

**Fig. 4:**
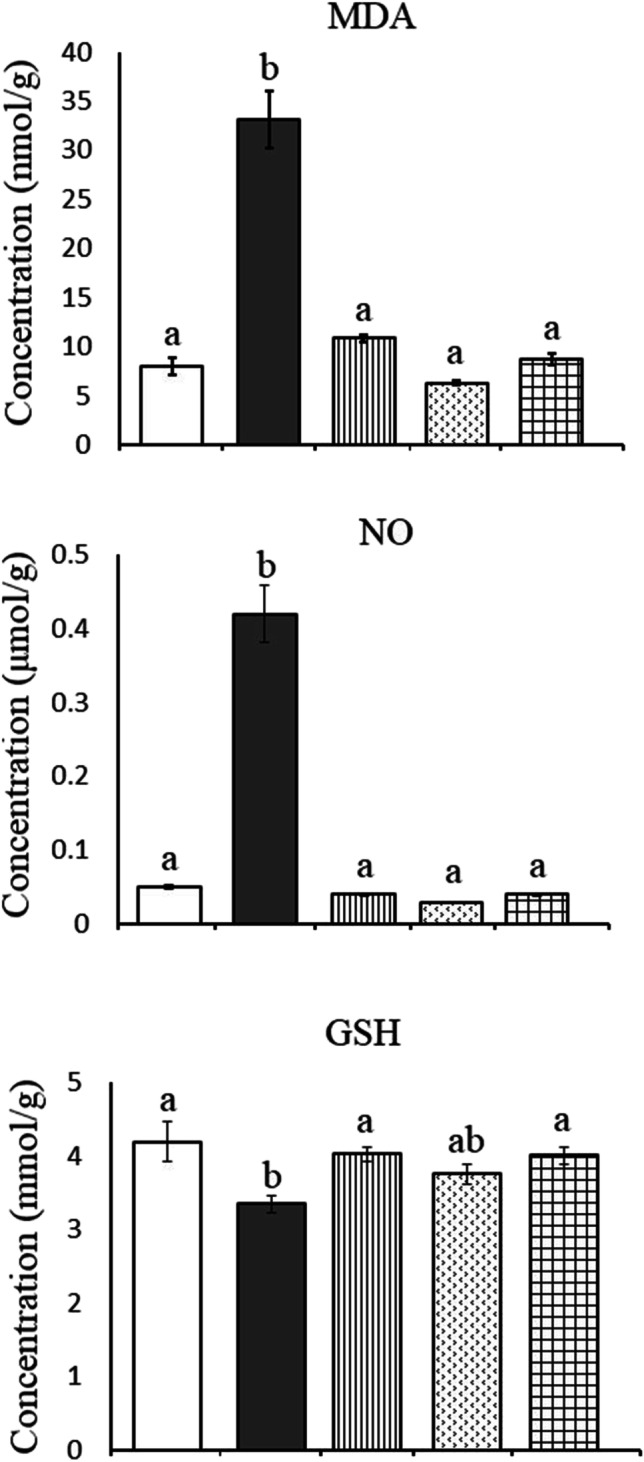
Effect of daily treatment with alpha-lipoic acid (ALA) (100 mg/kg) and/or caffeine-loaded chitosan nanoparticles (CAF-CS NPs) (20 mg/kg) for 30 days on the levels of malondialdehyde (MDA), nitric oxide (NO), and reduced glutathione (GSH) in the kidney of rat model of obesity induced by feeding rats on a high fat diet (HFD).  Control**.**  Rat model of obesity.  Rat model of obesity treated with ALA.  Rat model of obesity treated with CAF-CS NPs.  Rat model of obesity treated with ALA and CAF-CS NPs

In the present study, HFD induced a significant increase in hepatic MDA by + 131.89% (from 4.86 ± 0.74 to 11.27 ± 1.68, *P* = 0.002) and NO by + 550% (from 0.04 ± 0.006 to 0.26 ± 0.026, *P* = 0.000) in comparison to the control group. In the kidney of obese rats, a significant increase in the levels of MDA by + 314.38% (from 8.00 ± 0.84 to 33.15 ± 2.92, *P* = 0.000) and NO by + 740% (from 0.05 ± 0.003 to 0.42 ± 0.038, *P* = 0.000) was recorded as compared to the control group. These findings were associated with a significant decrease in GSH levels of liver (from 2.77 ± 0.13 to 2.36 ± 0.06, *P* = 0.038) and kidney (from 4.20 ± 0.27 to 3.35 ± 0.12, *P* = 0.005) recording − 14.80% and − 20.24%, respectively, less than the control values. Treatment with ALA and/or CAF-CS NPs restored the aforementioned-studied parameters to nearly control-like values except for a significant increase in hepatic MDA of rats treated with CAF-CS NPs (+ 71.81%) (Figs. 3 and 4).

### *Na*^+^*, K*^+^*-ATPase activity*

**Fig. 5:**
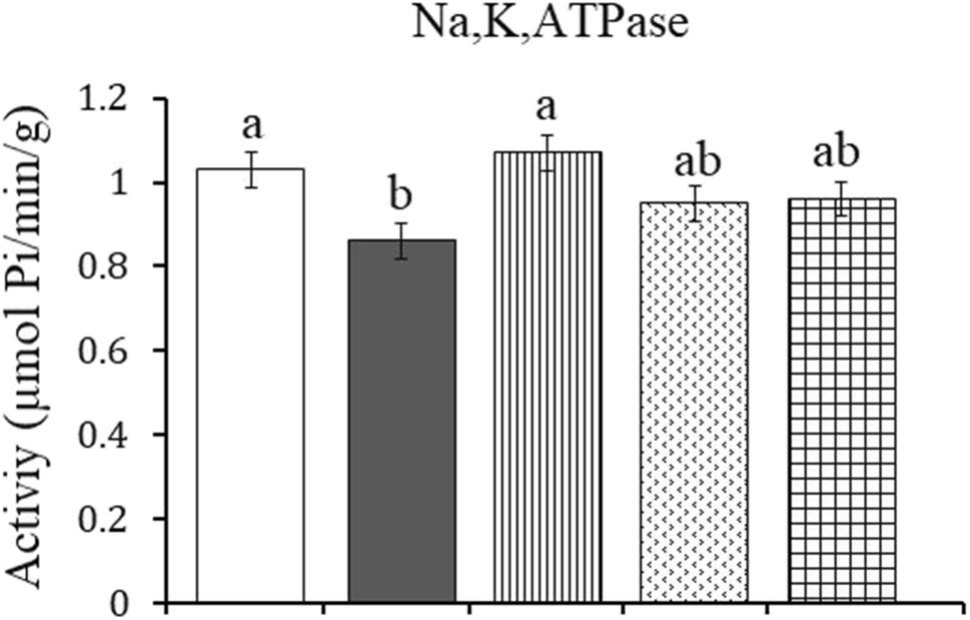
Effect of daily treatment with alpha-lipoic acid (ALA) (100 mg/kg) and/or caffeine-loaded chitosan nanoparticles (CAF-CS NPs) (20 mg/kg) for 30 days on the activity of Na^+^, k^+^-ATPase in the kidney of rat model of obesity induced by feeding rats on a high fat diet (HFD).  Control**.**  Rat model of obesity.  Rat model of obesity treated with ALA.  Rat model of obesity treated with CAF-CS NPs.  Rat model of obesity treated with ALA and CAF-CS NPs

As shown in Fig. 5, a significant decrease in renal Na^+^, K^+^-ATPase activity (from 1.03 ± 0.042 to 0.86 ± 0.035, *P* = 0.030) was observed in obese rats recording − 16.50% compared to the control values. Treatment with ALA succeeded in normalizing the significant decrease induced by HFD in Na^+^, K^+^-ATPase activity. However, treatment with CAF-CS NPs alone or in combination with ALA improves the activity of Na^+^, K^+^-ATPase which showed a nonsignificant change as compared to control and obese rats.

### Interleukin-1β and tumor necrosis factor-α

**Fig. 6:**
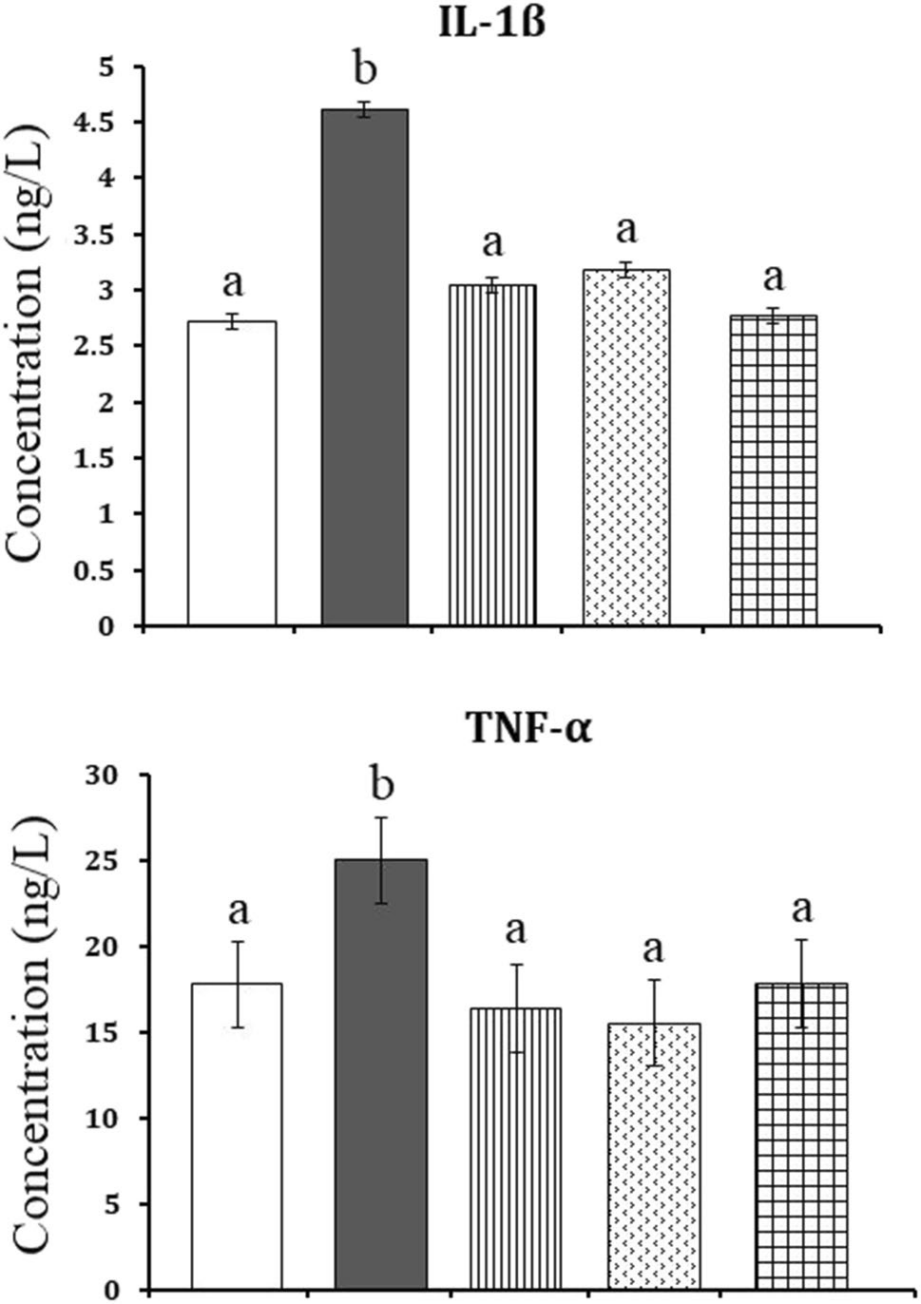
Effect of daily treatment with alpha-lipoic acid (ALA) (100 mg/kg) and/or caffeine-loaded chitosan nanoparticles (CAF-CS NPs) (20 mg/kg) for 30 days on the serum levels of interleukin-1β (IL-1β) and tumor necrosis factor-α (TNF-α) of rat model of obesity induced by feeding rats on a high fat diet (HFD).  Control**.**  Rat model of obesity.  Rat model of obesity treated with ALA.  Rat model of obesity treated with CAF-CS NPs.  Rat model of obesity treated with ALA and CAF-CS NPs

The present findings revealed that HFD significantly increased the serum levels of IL-1β by + 69.49% (from 2.72 ± 0.07 to 4.61 ± 0.26, *P* = 0.000) and TNF-α by + 40.72 (from 17.78 ± 2.53 to 25.02 ± 2.83,* P* = 0.026) as compared to the control values. Treatment with ALA and/or CAF-CS NPs succeeded in normalizing the increased levels of IL-1β and TNF-α induced by HFD (Fig. 6).

### Histopathological results

**Fig. 7:**
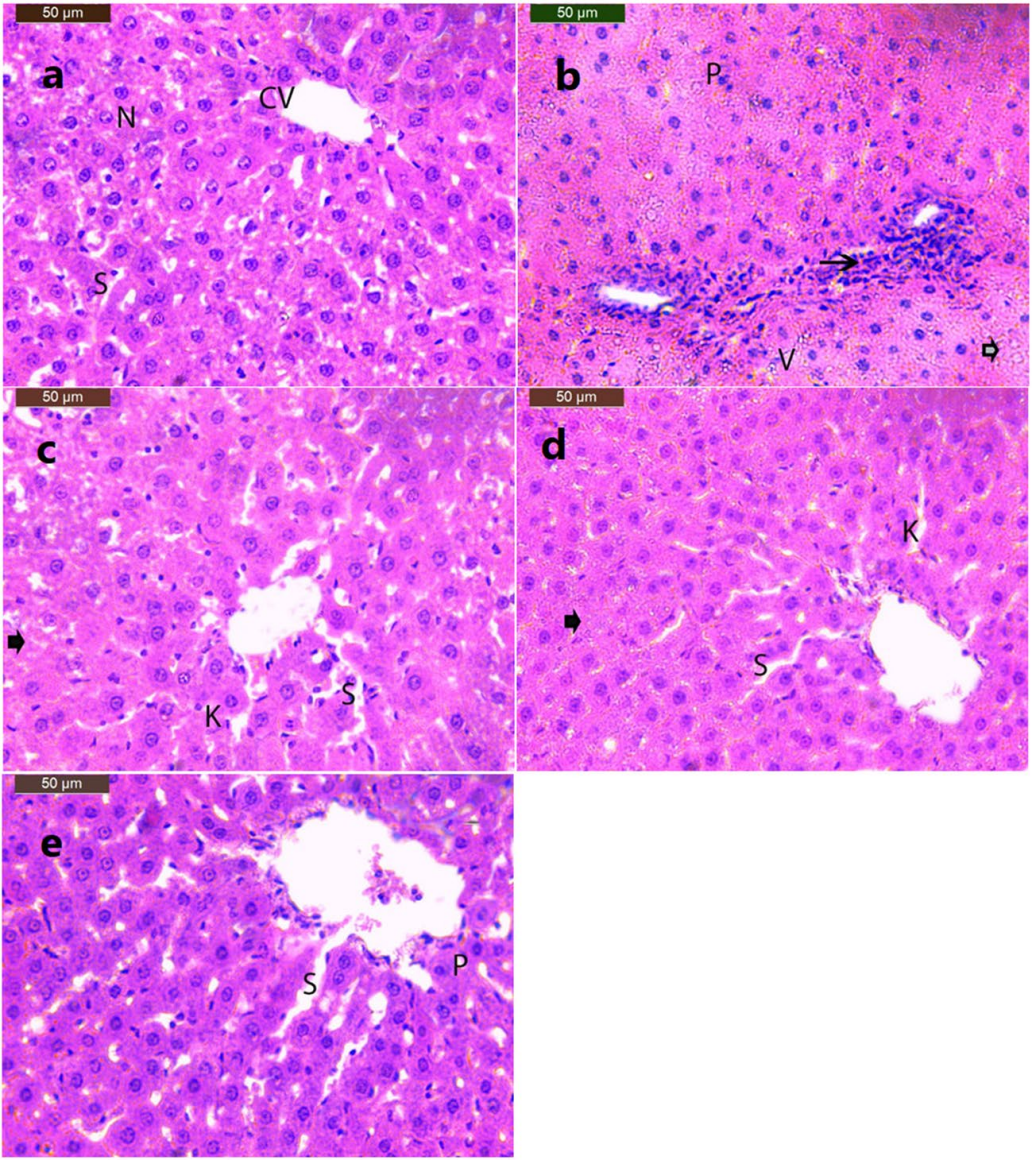
Effect of daily treatment with alpha-lipoic acid (ALA) (100 mg/kg) and/or caffeine-loaded chitosan nanoparticles (CAF-CS NPs) (20 mg/kg) for 30 days on the histopathological changes induced by obesity in the liver of rats (H&E × 200). **a** Photomicrograph of liver section of control rat showing normal hepatic architecture with central vein (CV), blood sinusoids (S), and prominent nuclei (N). **b** Section of the liver of obese rats showing disruption of normal architecture of hepatic lobules, hepatic necrosis (arrowhead), cytoplasmic vacuole (V) with lymphocytic infiltration around central vein and portal areas (arrow) and pyknotic nuclei. **c** Section of the liver of obese rats treated with ALA showing mild improvement with focal hepatocyte necrosis (arrowhead), inflammatory cells and mild activation of Kupffer cells (K). **d** Section of the liver of obese rats treated with CAF-CS NPs showing normal histological picture of the liver tissue with pericentral hepatic necrosis (arrowhead), dilated blood sinusoids (S) and mild activation of Kupffer cells (K). **e** Section of the liver of obese rats treated with ALA + CAF-CS NPs showing normal histological picture of the liver tissue with dilated blood sinusoids (S) and pyknotic nuclei (P)

The examined H&E-stained section of control livers showed a normal lobular architecture with central veins and radiating hepatic cords separated by narrow blood sinusoids and prominent nuclei (Fig. 7a). Liver sections of obese rats showed disruption of the normal architecture of hepatic lobules, hepatic necrosis, and cytoplasmic vacuoles with lymphocytic infiltration around the central vein and portal areas with pyknotic nuclei (Fig. 7b). Treatment with ALA induced mild improvement with focal hepatocyte necrosis, inflammatory cell infiltration, and mild activation of Kupffer cells (Fig. 7c). In obese rats treated with CAF-CS NPs, the liver tissue displayed a normal histological picture with pericentral hepatic necrosis, dilated blood sinusoids, and mild activation of Kupffer cells (Fig. 7d). Hepatic sections of obese rats treated with ALA and CAF-CS NPs combination showed improvement and nearly normalized hepatocytes with focal hepatocyte necrosis and inflammatory cells (Fig. 7e).

**Fig. 8:**
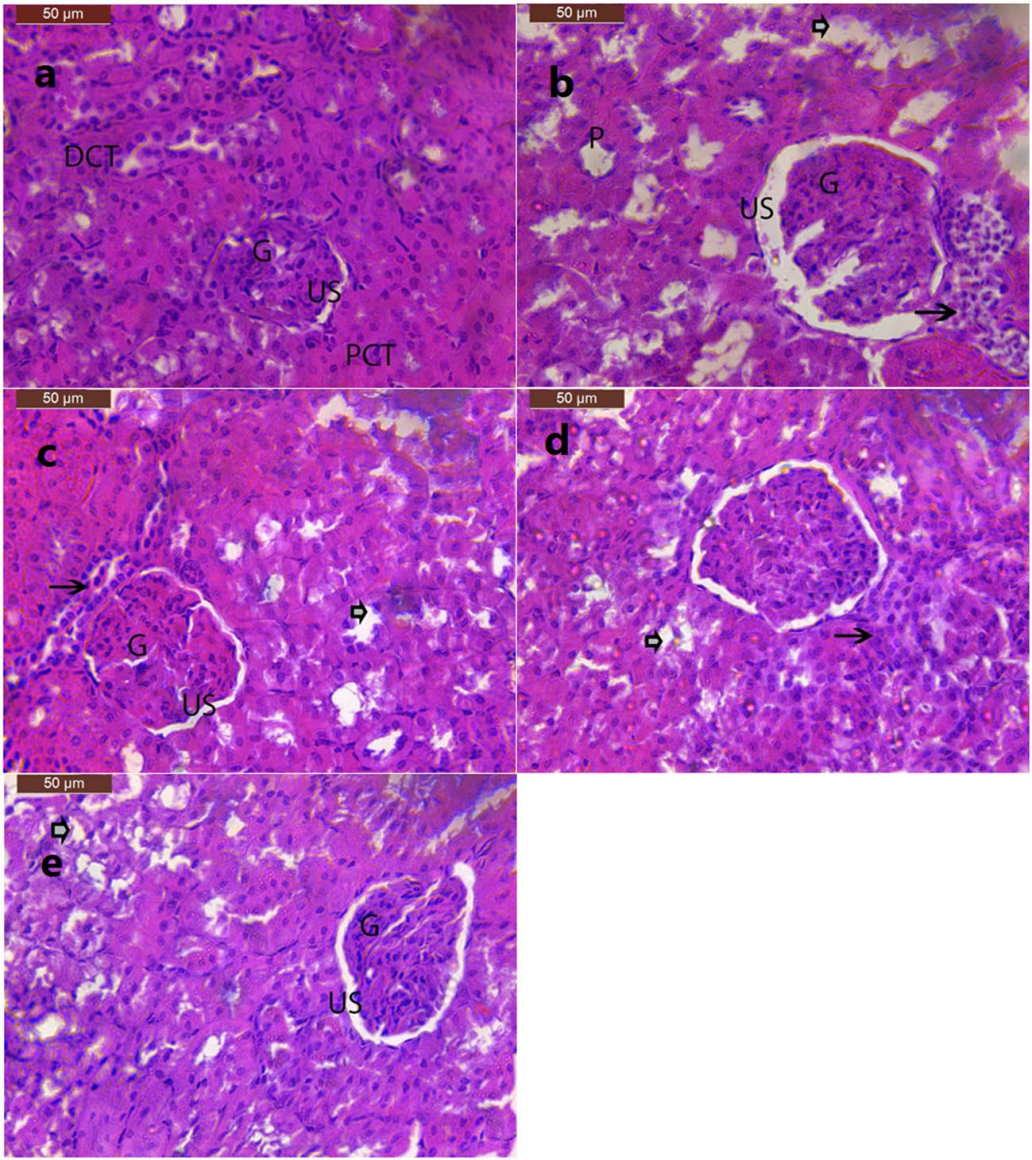
Effect of daily treatment with alpha-lipoic acid (ALA) (100 mg/kg) and/or caffeine-loaded chitosan nanoparticles (CAF-CS NPs) (20 mg/kg) for 30 days on the histopathological changes induced by obesity in the kidney of rats (H&E × 200). **a** Photomicrograph of kidney section of control rat showing normal structure of the glomerulus (G), urinary space (US), normal proximal convoluted tubules (PCT), and distal convoluted tubules (DCT). **b** Section of the kidney of obese rat showing degenerative changes in the glomerulus such as shrinkage (G) and widening of urinary space (US), degeneration of renal tubules, pyknotic nuclei (P) and interstitial inflammatory cells (arrow). **c** Section of the kidney of obese rats treated with ALA showing mild improvement in the architecture of kidney with mild dilatation of Bowman’s space (US), mild degenerated tubules (arrowhead) and pyknotic nuclei (arrow). **d** Section of the kidney of obese rats treated with CAF-CS NPs showing mild dilatation of Bowman’s space (US), mild degenerated tubules (arrowhead) and pyknotic nuclei (arrow). **e** Section of the kidney of obese rats treated with ALA + CAF-CS NPs showing almost normal architecture of the kidney with the exception of only few degenerated tubules (arrowhead) and pyknotic nuclei (P)

The histological examination of renal tissues of the control group revealed normal renal glomeruli surrounded by urinary space and normal proximal, distal, and convoluted tubules (Fig. 8a). In the renal tissue of rat model of obesity, there were degenerative changes in the glomeruli such as shrinkage and widening of urinary space. In addition, renal tubules revealed vacuolation and hydropic degeneration of epithelium, pyknotic nuclei, and interstitial inflammatory cells (Fig. 8b). Treatment with either ALA or CAF-CS NPs resulted in mild improvements in the architecture of the kidney. However, some glomeruli showed mild dilatation of Bowman’s space with mild degenerated tubules and pyknotic nuclei (Fig. 8c and d). Renal sections of rats treated with ALA in combination with CAF-CS NPs showed almost normal architecture of the kidney with the exception of a few degenerated tubules and pyknotic nuclei (Fig. 8e).

## Discussion

In the present study, the rat model of obesity was induced by feeding rats on a HFD for 20 weeks. Feeding rats on HFD is the most useful obesity model as it mimics the most common cause of obesity in humans. The obesity model was confirmed by measuring the BMI. A rat was considered obese when its BMI exceeded 0.68 g/cm^2^ (Novelli et al. 2007). The current results revealed that the HFD produced oxidative stress in the liver and kidney. This was evident from the significant increase in lipid peroxidation (MDA) and NO levels, and the significant decrease in GSH levels in hepatic and renal tissues. Beef tallow, which is rich in saturated fatty acids, was used as the source of fat in preparing the HFD in the current study. Overconsumption of saturated fatty acids leads to the accumulation of white adipose tissue; the liver continuously metabolizes the excess lipids, activating cytochrome P450 and resulting in overproduction of reactive oxygen species (ROS) (Kennedy et al. 2009; Lai et al. 2016). ROS in turn react with the cell membrane phospholipids, causing increased lipid peroxidation in hepatic tissues. Moreover, ROS and lipid peroxidation products either directly or indirectly harm the respiratory chain in hepatocytes causing oxidative damage to the mitochondrial genome. This in turn generates more ROS exacerbating the oxidative stress (Cichoż-Lach and Michalak 2014). The products of ROS and lipid peroxidation also activate stellate cells, leading to fibrosis (Carmiel-Haggai and Nieto 2005).

On the other hand, HFD was found to increase the activity of inducible nitric oxide synthase (iNOS) in the liver (Wan et al. 2000) and kidney (Chowdhury et al. 2022) of obese rats. This may explain the present elevated NO level in hepatic and renal tissues. At high level, NO induces cellular apoptosis by modulating both extrinsic and intrinsic signaling pathways in rat hepatocytes (Balakirev et al. 1997). NO can also combine with superoxide anion to form peroxynitrite (ONOO^−^), a powerful oxidant that can freely diffuse through intra- and intercellular pathways (Stamler et al. 1992). The accumulation of ONOO^−^ interacts with lipids, DNA, and proteins causing irreversible cellular damage (Pacher et al. 2007).

The decreased GSH, which acts as an important endogenous antioxidant, may also potentiate the oxidative and nitrosative stress induced by HFD in the present study. GSH acts directly by scavenging free radicals and indirectly as a substrate for some antioxidant enzymes (Birk et al. 2013). As a result, the current reduction in hepatic and renal GSH level could be due to its consumption in scavenging the free radicals generated by HFD.

The oxidative and nitrosative stress induced by HFD may mediate the histopathological disruption of the normal architecture of hepatic lobules, hepatic necrosis, and cytoplasmic vacuole with lymphocytic infiltration (steatohepatitis) around the central vein and portal areas with pyknotic nuclei that have been reported in the present study. In agreement with our findings, Cui et al. (2011) found that HFD induced excessive production of ROS and increased lipid peroxidation, thereby causing degradation of hepatocyte membranes and cellular leakage of liver enzymes into the plasma. This may explain the present increased AST, ALT, and ALP activities in the serum of obese rats. Under normal conditions, these enzymes are found in high concentrations in the cytoplasm of hepatocytes, and they are released into the circulation during hepatic damage (Contreras-Zentella and Hernández-Muñoz 2016).

It has been reported that long-term consumption of a HFD alters renal lipid metabolism by inducing an imbalance between lipogenesis and lipolysis in the kidney resulting in renal lipid accumulation and lipid peroxidation which has been correlated with a progressive decline in renal function (Jiang et al. 2005; Guebre-Egziabher et al. 2013). Moreover, adipose tissue accumulates around the kidneys and enters the medullary sinuses, increasing intrarenal pressures and causing renal tissue damage (Kume et al. 2007). The present findings showed that HFD induced oxidative stress in renal tissue of obese rats as indicated by the elevated MDA and NO and the reduced GSH levels. Nephrons are rich in mitochondria and HFD has been found to promote renal injury by inducing mitochondrial dysfunction causing overproduction of ROS and oxidative stress which leads to renal tubular cell apoptosis (Sun et al. 2020). This, in turn, results in a decrease in the glomerular filtration rate and an accumulation of urea and creatinine in the blood (Baracho et al. 2016). This may explain the elevated serum levels of urea and creatinine in the present model of obesity. Moreover, oxidative stress and nitrosative stress induced by HFD consumption may also be responsible for the reduced renal Na^+^, K^+^-ATPase activity in the present study. Na^+^, K^+^-ATPase is highly sensitive to oxidative stress (Dobrota et al. 1999). The present findings agree with the study of Briffa et al. (2015) who reported that HFD can cause a reduction in Na^+^, K^+^-ATPase expression which could also potentially impact albumin uptake and sodium reabsorption. Normal Na^+^, K^+^-ATPase activity is essential for proper kidney function. The importance of the Na^+^, K^+^-ATPase pump for the kidneys comes from its great number, which reaches up to 50 million pumps per cell in the distal convoluted tubule (El Mernissi and Doucet 1984). Moreover, Na^+^, K^+^-ATPase induces important physiological roles in the kidneys, with a primary function in Na^+^ and water reabsorption, which is fundamental for keeping body fluid and electrolyte homeostasis (Reinhard et al. 2013). As a result, the reduced Na^+^, K^+^-ATPase activity caused by HFD may contribute to the impairment of renal function observed in the current study.

The adverse effect of obesity on the kidney was also manifested by the histopathological changes which included shrinkage of the glomerulus, widening of the urinary space, vacuolation of renal tubules, hydropic degeneration of renal tubular epithelium, pyknosis of the nuclei, and inflammatory cell infiltration. These changes could be attributed to the oxidative and nitrosative stress and the reduced Na^+^, K^+^-ATPase activity induced by HFD. It has been reported that excessive renal lipid deposition can result in renal tubular cell injury (Nosadini and Tonolo 2011), tubulointerstitial fibrosis (Takabatake et al. 2017), podocyte damage, mesangial sclerosis (Abrass 2004), and structural glomeruli alterations (Keane 2000; Zhou et al. 2016).

Obesity is a chronic low-grade systemic inflammatory state characterized by increased pro-inflammatory cytokine secretion from adipose tissue and infiltration of leukocytes, including macrophages, into adipose tissue. This chronic inflammation contributes to the development of metabolic disorders like non-alcoholic fatty liver disease and chronic kidney disease (Schäffler et al. 2007). The present study clearly revealed a significant increase in serum levels of pro‑inflammatory cytokines like tumor necrosis factor-α (TNF-α) and interleukin-1β (IL-1β) in obese rats indicating the development of severe inflammation. These results agree with the study of Cortez et al. (2013) who found that HFD induced a significant increase in TNF-α and IL-1β levels by increasing the gene expression of nuclear transcription factor kappa B (NF-κB). Moreover, obesity induced progressive and cumulative cell injury caused by the large body mass’s pressure. Cell injury leads to the release of pro‑inflammatory cytokines, which stimulate the production of ROS from the tissues (Khan et al. 2006).

The present data clearly revealed that obesity produced lipotoxicity. This term is used to describe the deleterious effects exerted by lipids on cells and tissues (Martins and Mas 2015; Escasany et al. 2019).

The present data showed that alpha-lipoic acid (ALA) attenuated the impairment of hepatic and renal functions induced in obese rats. This was demonstrated by the ability of ALA to normalize the activities of AST, ALT, and ALP (liver functions) as well as the levels of urea and creatinine (kidney functions). Our results are in parallel with previous studies reporting that ALA attenuated the hepatic and renal toxicities induced by cyclophosphamide and colistin (Abdul-Hamid et al. 2020; Oktan et al. 2021). This ameliorative effect could be attributed to its ability to prevent the oxidative stress induced by HFD. It has been demonstrated that ALA acts as a potent antioxidant by scavenging reactive oxygen and nitrogen species (Bast and Haenen 2003), increasing the activities of antioxidant enzymes (Gomes and Negrato 2014), and reducing the oxidized antioxidants like glutathione, vitamin C, and vitamin E (Rochette et al. 2015). Moreover, ALA can increase GSH synthesis by increasing the availability of cysteine in cells through the conversion of cystine to cysteine (Han et al. 1997). This may explain the ability of ALA to prevent the increase in lipid peroxidation and NO and the reduced level of GSH induced by HFD in the present study. In addition, ALA reduced the production of NO by suppressing the activity and expression of iNOS (Demarco et al. 2004; Tanaka et al. 2015).

Besides the potent antioxidant activity, ALA attenuated the inflammation induced in rat model of obesity. This was evident from its ability to restore the elevated TNF-α and IL-1β levels induced by HFD. The present data agree with the study of Çakır et al. (2015) who revealed that ALA decreased the serum levels of TNF-α and IL-1β in rats. The anti-inflammatory effect of ALA could be mediated by its ability to inhibit the release of pro-inflammatory cytokines that participate in inflammatory signaling by reducing the gene expression of NF-κB occurring in HFD-fed rats (Sztolsztener et al. 2022). The antioxidant and anti-inflammatory effects of ALA could have a role in minimizing the histopathological alterations induced in the liver and kidney of obese rats. In addition, the restored renal Na^+^, K^+^-ATPase activity induced by ALA in the present study may result in maintaining body fluid and electrolyte homeostasis. This effect could also contribute to reduced renal histopathological changes. The recovered renal Na^+^, K^+^-ATPase activity may be attributed to ALA’s antioxidant effect and its stimulatory effect on the biosynthesis of ATP which is the main substrate for Na^+^, K^+^-ATPase (Shay et al. 2009).

The present findings also revealed that CAF-CS NPs ameliorated the hepatic and renal alterations induced in obese rats. Treatment with CAF-CS NPs successfully restored ALP, ALT, and AST. This effect may be due to the ability of caffeine to maintain hepatocyte membrane integrity, thereby preventing the leakage of hepatic enzymes from hepatocytes to the systemic circulation. Caffeine consumption, on the other hand, has been shown to have a renal protective effect against chronic kidney diseases by increasing glomerular filtration rate and maintaining the renin-angiotensin system (Kennedy et al. 2020; Srithongkul and Ungprasert 2020). This may explain the restored urea and creatinine levels induced by caffeine in the present study.

The hepato‑renal protective effect of CAF-CS NPs in the present study could be attributed to their ability to attenuate oxidative stress and inflammation induced by HFD. CAF-CS NPs reduced the increased MDA in the liver and kidney. This effect may be due to the action of caffeine as a free radical scavenger (León-Carmona and Galano 2011). Thus, caffeine could scavenge oxygenated free radicals and NO radicals preventing the formation of peroxynitrite. In addition, the increased GSH content in the liver and kidney by CAF-CS NPs may be due to the stimulatory effect of caffeine on the cellular synthesis of GSH (Aoyama et al. 2011). The reported suppressive effect of caffeine on the gene expression of iNOS (de Alcântara Almeida et al. 2021) may enable CAF-CS NPs to restore NO levels in the liver and kidney of obese rats. CAF-CS NPs also demonstrated an anti-inflammatory effect, as evident from their ability to restore elevated TNF-α and IL-1β to control levels by inhibiting the production of pro-inflammatory cytokines such as TNF-α and IL-1β via the cyclic adenosine monophosphate/protein kinase A (cAMP/PKA) pathway (Horrigan et al. 2004).

The current data show that CAF-CS NPs reduced hepatic and renal histopathological changes caused by obesity. However, pericentral hepatic necrosis with dilated blood sinusoids and mild activation of Kupffer cells in hepatic sections were observed. Also, treatment with CAF-CS NPs reduced the degeneration of renal tubules and the dilatation of Bowman’s space caused by HFD in kidney. The present improvement in the histopathological picture in rats treated with CAF-CS NPs could be attributed to the antioxidant and anti-inflammatory activities of CAF-CS NPs. The improved Na^+^, K^+^-ATPase activity induced by CAF-CS NPs in the renal tissue may play a role in restoring renal function and reducing renal histopathology in obese rats.

The present study extended to investigate the combined administration of ALA + CAF-CS NPs on the hepatic and renal complications induced by obesity. Treatment of obese rats with ALA + CAF-CS NPs restored the hepatic and renal functions. In addition, they ameliorated the increased levels of lipid peroxidation and NO levels and the reduced level of GSH in the liver and kidney. Moreover, the combined administration of ALA + CAF-CS NPs showed anti-inflammatory effect as they suppressed the elevated levels of TNF-α and IL-1β induced in the sera of obese rats. The antioxidant and anti-inflammatory effects together with the restored Na^+^, K^+^-ATPase obtained by ALA + CAF-CS NPs in the present study may have a substantial role in improving the histopathological alterations induced in hepatic and renal tissues of obese rats. The present findings showed that the co-treatment with ALA and CAF-CS NPs improved almost all the histopathological changes resulting from obesity in hepatic and renal tissues. However, focal hepatocyte necrosis with mild infiltration of inflammatory cells and a few degenerated tubules and pyknotic nuclei were still observed in the liver and kidney, respectively.

The results obtained in the present study indicated that ALA and/or CAF-CS NPs reduced obesity as indicated by the BMI which recorded the least measure when obese rats were co-treated with ALA and CAF-CS NPs indicating a synergistic effect against obesity.

## Conclusion

According to the present data, it could be concluded that ALA and/or CAF-CS NPs attenuated the hepatic and renal complications induced by obesity in rats. This effect could be attributed to their ability to reduce BMI, oxidative stress, and inflammation induced by feeding rats on HFD. Based on the present findings, ALA and/or CAF-CS NPs are recommended as anti-obesity agents in human.

## Data Availability

The authors confirm the availability of all required data and materials.
